# Association of preoperative prognostic nutritional index with postoperative delirium after gastric cancer surgery

**DOI:** 10.3389/fnut.2026.1817605

**Published:** 2026-05-20

**Authors:** Xiaoya Hong, Jiayu Wang

**Affiliations:** Department of Anesthesiology, The Affiliated Huaian No. 1 People’s Hospital of Nanjing Medical University, Huaian, Jiangsu, China

**Keywords:** biomarker, clinical nutrition, gastric cancer, postoperative delirium, prognostic nutritional index

## Abstract

**Background:**

Postoperative delirium (POD) is a common complication following gastric cancer (GC) surgery, leading to increased morbidity, prolonged hospitalization, and higher healthcare costs. Although other clinical and inflammatory markers have shown promise, the prognostic nutritional index (PNI)—a marker reflecting both immune and nutritional status—has rarely been evaluated for its ability to predict POD among GC patients. This study aimed to determine whether preoperative PNI could serve as an independent predictor of POD in GC cases undergoing gastrectomy.

**Methods:**

In our retrospective observational study, we analyzed relevant data from 354 GC patients after gastrectomy between January 2023 and December 2024. Receiver operating characteristic (ROC) analysis was used to ascertain the predictive value of PNI for POD. Univariate and multivariate logistic regression analyses were performed to identify independent risk factors for POD.

**Results:**

Of 354 GC patients, 103 (29.1%) developed POD after gastrectomy. Compared to patients without POD, those who developed POD had significantly higher proportions of older age (>60 years), longer hospital stay time (≥19 days), lower platelet counts (≥206*10^9^/L), ASA III, lower lymphocyte counts (≤1.45*10^9^/L), low albumin levels (<41.1 g/L), and low PNI (≤48.4). ROC analysis indicated that preoperative PNI predicted POD with an AUC of 0.780 at a cut-off value of 48.4, a sensitivity of 82.52%, a specificity of 65.34%, and a Youden index of 0.4786. Univariate logistic regression analysis revealed that age ≥60 years, hospital stay time ≥19 days, platelet count ≥206*10^9^/L, ASA III, lymphocyte count ≤1.45*10^9^/L, albumin <41.1 g/L, and preoperative PNI ≤ 48.4 were associated with the risk of POD. Multivariate logistic regression analysis confirmed that age ≥60 years, ASA III, and preoperative PNI ≤ 48.4 (OR = 3.952, 95% CI: 1.742–8.964, *p* = 0.001) were independent risk factors of POD.

**Conclusion:**

Preoperative PNI is an independent and clinically useful predictor of POD in GC patients. Regular PNI screening may help identify at-risk individuals among GC patients.

## 1 Introduction

Gastric cancer (GC) is one of leading causes of global cancer-related morbidity and mortality, with high incidence rates observed particularly in Eastern Europe, East Asia, and South America (1, 2). A study showed that China recorded the highest incidence, prevalence, and mortality of GC patients in East Asian countries in both 1990 and 2021 (3). Annually, approximately one million new GC cases are diagnosed worldwide, resulting in over 650,000 deaths (4). High salt intake, *Helicobacter pylori* infection, smoking, obesity, and genetic predisposition are recognized risk factors for GC (2). Despite advances in surgical techniques and perioperative management, GC gastrectomy still bears a considerable burden of postoperative complications (5, 6). Among these, postoperative delirium (POD) was a common complication and it was considered a major and common neuropsychiatric syndrome, which has impacts on patient recovery, hospitalization extensions, medical costs as well as mortality (7, 8). The POD incidence for GC patients ranged from 4.1 to 51.0% (9, 10). Delirium is characterized by acute changes in awareness, attention, and cognition. Its pathophysiology is multifactorial, involving inflammatory responses, metabolic disturbances, neuroendocrine dysregulation, oxidative stress, and nutritional status (11, 12).

The prognostic nutritional index (PNI) is calculated from serum albumin levels and total lymphocyte count. It has gained attention as a composite marker reflecting both nutritional and immunological status. Multiple studies have demonstrated that PNI was related to the risk of postoperative complications, and the survival in GC cases (13–19). In addition, studies reported that PNI was a risk factor for POD in colorectal cancer (CRC) (20), and esophageal cancer (21, 22). However, the association between preoperative PNI and POD in GC patients has not been specifically investigated. To address this issue, this study aimed to evaluate whether preoperative PNI was independently associated with the risk of POD among GC patients. The identification of an accessible, non-invasive, and economical marker like PNI could assist clinicians in stratifying POD risk in high-risk surgical GC patients.

## 2 Methods

### 2.1 Study design and participants

This retrospective observational study was performed in Huaian No. 1 People’s Hospital. GC patients who underwent gastrectomy were included in this study between January 2023 and December 2024. The inclusion criteria included: (1) histologically confirmed GC with TNM stage I–III; (2) age between 18 and 90 years; (3) cases undergoing gastrectomy. Exclusion criteria were as follows: (1) preoperative diagnosis of malnutrition; (2) use of antipsychotic medications prior to surgery; (3) postoperative infections; (4) history of mental illness; (5) incomplete clinical data. All GC patients provided the written informed consent. This study was in accordance with the Declaration of Helsinki guidelines. This study was approved by the Ethics committee of our hospital (Approval No. KY-2025-150-01).

### 2.2 Data collection

Clinical and demographic data were collected, including sex, age, comorbidities (diabetes and hypertension), tumor node metastasis (TNM) stage, surgical type, extent of gastric resection, hospital stay time, operative time, and anesthesia time. Preoperative laboratory values—neutrophil, white blood cell (WBC), lymphocyte, monocyte, and platelet counts, as well as serum albumin—were also recorded. The calculation formula of PNI was as follows: PNI = albumin (g/L) + 5 * lymphocyte count (10⁹/L).

### 2.3 Outcome measurement

The diagnosis of POD was according to the criteria of Confusion Assessment Method (CAM) (23). Specific diagnostic criteria of CAM were shown in our previous study (21). POD was evaluated twice every day from postoperative day 1 to postoperative day 7.

### 2.4 Statistical analysis

Continuous variables were displayed as mean ± standard deviation or median, and categorical variables were shown as frequencies (percentages). For continuous variables, the independent t-test or Mann–Whitney U test was applied as appropriate. For categorical variables, the chi-square test or Fisher’s exact test was used. The predictive performance of PNI for POD was assessed using receiver operating characteristic (ROC) curve analysis. Univariate and multivariate logistic regression analyses were conducted to determine the independent factors associated with POD, with results reported as 95% confidence intervals (CIs) and odds ratios (ORs). All relevant analyses were conducted using SPSS version 22.0 and MedCalc, with a two-tailed *p* < 0.05 considered statistically significant.

## 3 Results

### 3.1 Patient characteristics

**Figure 1:**
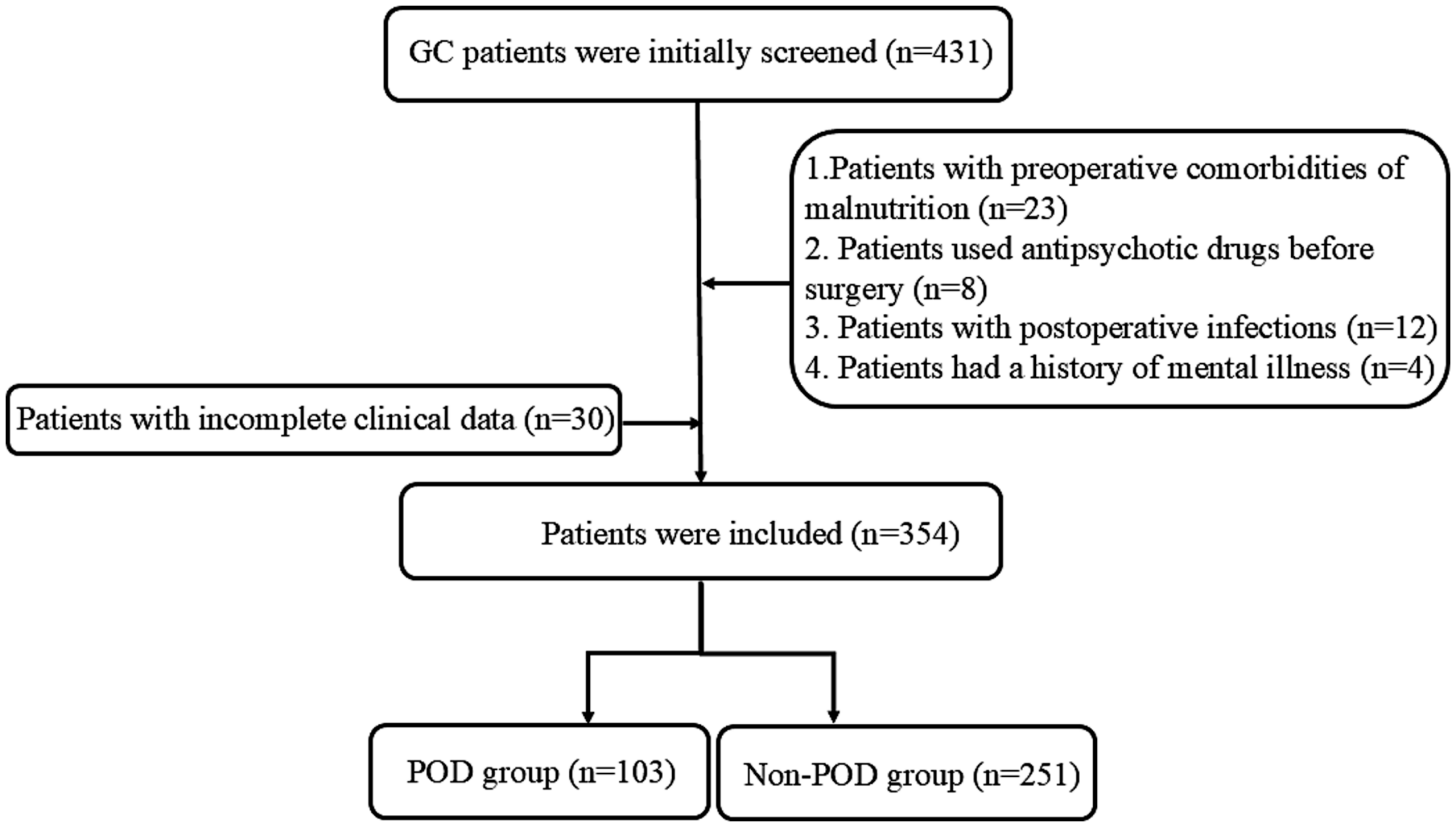
Flow diagram of enrolled patients.

This study included 354 patients who underwent GC surgery, among whom 103 (29.1%) developed POD and 251 did not (Figure 1). Table 1 summarizes the clinical and laboratory features of the two groups. Several significant differences were shown between the delirium and non-delirium groups. Patients aged ≥60 years were more prevalent in the delirium group (88.3%) than in the non-delirium group (62.2%) (*p* < 0.001). Regarding hospital stay time, hospital stay time ≥19 days was more common in the delirium group than in the non-delirium group (66.4% vs. 51.8%, *p* = 0.035). In addition, delirium group showed a higher percentage of ASA III than the non-delirium group (52.4% vs. 25.1%, *p* < 0.001). With respect to laboratory markers, patients with POD showed significantly higher proportions of lymphocyte count ≤1.45 × 10⁹/L (66.0% vs. 43.8%, *p* < 0.001) and platelet count <206*10^9^/L (59.2% vs. 46.6%, *p* = 0.031), and level of albumin <41.1 g/L (76.7% vs. 40.6%, *p* < 0.001) than patients with non-POD. A lower PNI ≤ 48.4 was significantly more common in the delirium group (77.7%) compared to the non-delirium group (36.7%) (*p* < 0.001). No significant differences were reported between the two groups regarding sex, diabetes mellitus, hypertension, diabetes mellitus, TNM stage, types of surgery, degree of gastric resection, WBC count, neutrophil count, monocyte count, surgery time, and anesthesia time.

**Table 1:** Clinicopathological characteristics of gastric cancer patients between non-delirium and delirium groups.

Variables	Non-delirium group (*n* = 251)	Delirium group (*n* = 103)	*p*-value
Sex, *n* (%)			0.293
Female	67 (26.7%)	22 (21.4%)	
Male	184 (73.3%)	81 (78.6%)	
Age, *n* (%)			<0.001^#^
≤60 years	95 (37.8%)	12 (11.7%)	
>60 years	156 (62.2%)	91 (88.3%)	
Hypertension, *n* (%)			0.416
No	182 (72.5%)	79 (76.7%)	
Yes	69 (27.5%)	24 (23.3%)	
Diabetes mellitus, *n* (%)			0.520
No	234 (93.2%)	94 (91.3%)	
Yes	17 (6.8%)	9 (8.7%)	
TNM stage, *n* (%)			0.105
I-II	155 (61.8%)	54 (52.4%)	
III	96 (38.2%)	49 (47.6%)	
Types of surgery, *n* (%)			0.103
Laparoscopic surgery	231 (92.0%)	89 (86.4%)	
Open surgery	20 (8.0%)	14 (13.6%)	
Degree of gastric resection, *n* (%)			0.093
Partial gastrectomy	144 (57.4%)	49 (47.6%)	
Total gastrectomy	107 (42.6%)	54 (52.4%)	
Hospital stay time, *n* (%)			0.035^#^
<19 days	121 (48.2%)	37 (35.9%)	
≥19 days	130 (51.8%)	66 (64.1%)	
WBC, *n* (%)			0.777
<5.38*10^9^/L	125 (49.8%)	53 (51.5%)	
≥5.38*10^9^/L	126 (50.2%)	50 (48.5%)	
Neutrophil, *n* (%)			0.558
<3.37*10^9^/L	128 (51.0%)	49 (47.6%)	
≥3.37*10^9^/L	123 (49.0%)	54 (52.4%)	
Monocyte, *n* (%)			0.379
<0.38*10^9^/L	125 (49.8%)	46 (44.7%)	
≥0.38*10^9^/L	126 (50.2%)	57 (55.3%)	
Platelet, *n* (%)			0.031^#^
<206*10^9^/L	117 (46.6%)	61 (59.2%)	
≥206*10^9^/L	134 (53.4%)	42 (40.8%)	
ASA score, *n* (%)			<0.001^#^
I-II	188 (74.9%)	49 (47.6%)	
III	63 (25.1%)	54 (52.4%)	
Surgery time, *n* (%)			0.121
<3.36 h	130 (51.8%)	44 (42.7%)	
≥3.36 h	121 (48.2%)	59 (57.3%)	
Anesthesia time, *n* (%)			0.092
<3.63 h	132 (52.6%)	44 (42.7%)	
≥3.63 h	119 (47.4%)	59 (57.3%)	
Lymphocyte, *n* (%)			<0.001^#^
>1.45*10^9^/L	141 (56.2%)	35 (34.0%)	
≤1.45*10^9^/L	110 (43.8%)	68 (66.0%)	
Albumin, *n* (%)			<0.001^#^
≥41.1 g/L	149 (59.4%)	24 (23.3%)	
<41.1 g/L	102 (40.6%)	79 (76.7%)	
PNI, *n* (%)			<0.001^#^
>48.4	159 (63.3%)	23 (22.3%)	
≤48.4	92 (36.7%)	80 (77.7%)	

TNM stage, tumor node metastasis stage; WBC, white blood cell; ASA, American Society of Anesthesiologists; PNI, prognostic nutritional index. ^#^Indicating statistically significant (*p* < 0.05).

### 3.2 Predictive value of preoperative PNI for POD

**Figure 2:**
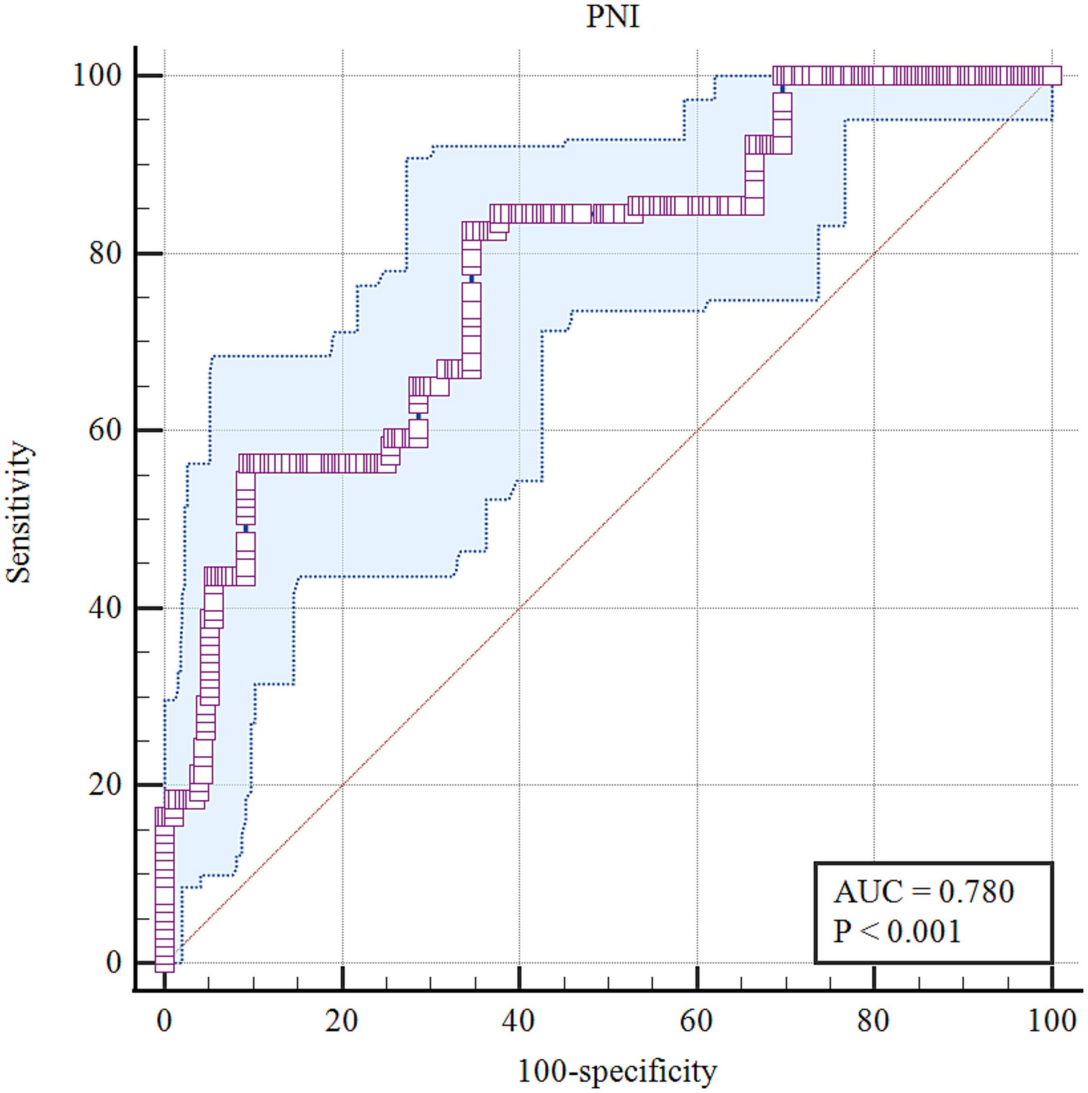
The receiver operating characteristic curve analysis of preoperative PNI for POD.

ROC analysis was used to evaluate the predictive ability of preoperative PNI for POD (Figure 2). The optimal cut-off value of PNI was 48.4, with a sensitivity of 82.52%, and a specificity of 65.34%, and an AUC value of 0.780 (0.733–0.822). The Youden index was 0.4786 (Table 2). Overall, preoperative PNI demonstrated moderate predictive ability for POD.

**Table 2:** Optimal cut-off value of PNI for predicting the postoperative delirium.

Variables	Cut-off value	Sensitivity %	Specificity %	AUC value	*p* value	Youden index
PNI	*≤*48.4	82.52	65.34	0.780(0.733–0.822)	<0.001	0.4786

PNI, prognostic nutritional index.

### 3.3 Independent risk factors for POD

Univariate logistic regression analysis identified several factors associated with POD (Table 3), including age ≥60 years (OR = 4.618, 95% CI: 2.402–8.879, *p* < 0.001), hospital stay time ≥19 days (OR = 1.660, 95% CI: 1.035–2.663, *p* = 0.035), platelet count ≥206 × 10⁹/L (OR = 0.601, 95% CI: 0.378–0.957, *p* = 0.032), ASA III classification (OR = 3.289, 95% CI: 2.034–5.318, *p* < 0.001), lymphocyte count ≤1.45 × 10⁹/L (OR = 2.490, 95% CI: 1.544–4.016, *p* < 0.001), albumin <41.1 g/L (OR = 4.808, 95% CI: 2.854–8.101, *p* < 0.001), and preoperative PNI ≤ 48.4 (OR = 6.011, 95% CI: 3.538–10.213, *p* < 0.001). However, sex, TNM stage, hypertension, diabetes mellitus, types of surgery, degree of gastric resection, WBC count, neutrophil count, monocyte count, surgery time, and anesthesia time were not associated with the risk of POD in univariate analysis. Multivariate logistic regression analysis confirmed that age ≥60 years (OR = 3.894, 95% CI: 1.894–8.003, *p* < 0.001), ASA III (OR = 2.433, 95% CI: 1.386–4.271, *p* = 0.002), and preoperative PNI ≤ 48.4 (OR = 3.952, 95% CI: 1.742–8.964, *p* = 0.001) were independent risk factors for POD (Table 4). However, hospital stay time, lymphocyte count, platelet count, and albumin level were not risk factors for POD.

**Table 3:** Univariate logistic regression analyses for predicting postoperative delirium.

Variables	OR (95% CI)	*p*-value
Sex, *n* (%)		
Female	Reference	
Male	1.341 (0.775, 2.319)	0.294
Age, *n* (%)		
≤60 years	Reference	
>60 years	4.618 (2.402, 8.879)	<0.001^#^
Hypertension, *n* (%)		
No	Reference	
Yes	0.801 (0.470, 1.367)	0.416
Diabetes mellitus, *n* (%)		
No	Reference	
Yes	1.318 (0.567, 3.061)	0.521
TNM stage, *n* (%)		
I-II	Reference	
III	1.465 (0.922, 2.328)	0.106
Types of surgery, *n* (%)		
Laparoscopic surgery	Reference	
Open surgery	1.817 (0.880, 3.753)	0.107
Degree of gastric resection, *n* (%)		
Partial gastrectomy	Reference	
Total gastrectomy	1.483 (0.936, 2.351)	0.093
Hospital stay time, *n* (%)		
<19 days	Reference	
≥19 days	1.660 (1.035, 2.663)	0.035^#^
WBC, *n* (%)		
<5.38*10^9^/L	Reference	
≥5.38*10^9^/L	0.936 (0.592, 1.481)	0.777
Neutrophil, *n* (%)		
<3.37*10^9^/L	Reference	
≥3.37*10^9^/L	1.147 (0.725, 1.815)	0.559
Monocyte, *n* (%)		
<0.38*10^9^/L	Reference	
≥0.38*10^9^/L	1.229 (0.776, 1.948)	0.380
Platelet, *n* (%)		
<206*10^9^/L	Reference	
≥206*10^9^/L	0.601 (0.378, 0.957)	0.032^#^
ASA score, *n* (%)		
I-II	Reference	
III	3.289 (2.034, 5.318)	<0.001^#^
Surgery time, *n* (%)		
<3.36 h	Reference	
≥3.36 h	1.441 (0.907, 2.287)	0.122
Anesthesia time, *n* (%)		
<3.63 h	Reference	
≥3.63 h	1.487 (0.937, 2.362)	0.092
Lymphocyte, *n* (%)		
>1.45*10^9^/L	Reference	
≤1.45*10^9^/L	2.490 (1.544, 4.016)	<0.001^#^
Albumin, *n* (%)		
≥41.1 g/L	Reference	
<41.1 g/L	4.808 (2.854, 8.101)	<0.001^#^
PNI, *n* (%)		
>48.4	Reference	
≤48.4	6.011 (3.538, 10.213)	<0.001^#^

TNM stage, tumor node metastasis stage; WBC, white blood cell; ASA, American Society of Anesthesiologists; PNI, prognostic nutritional index. ^#^ Indicating statistically significant (*p* < 0.05).

**Table 4:** Multivariate logistic regression analyses for predicting postoperative delirium.

Variables	OR (95% CI)	*p*-value
Age, *n* (%)		
≤60 years	Reference	
>60 years	3.894 (1.894, 8.003)	0.000^#^
Hospital stay time, *n* (%)		
<19 days	Reference	
≥19 days	1.088 (0.622, 1.903)	0.768
Platelet, *n* (%)		
<206*10^9^/L	Reference	
≥206*10^9^/L	0.780 (0.453, 1.345)	0.372
ASA score, *n* (%)		
I-II	Reference	
III	2.433 (1.386, 4.271)	0.002^#^
Lymphocyte, *n* (%)		
>1.45*10^9^/L	Reference	
≤1.45*10^9^/L	1.535 (0.851, 2.768)	0.155
Albumin, *n* (%)		
≥41.1 g/L	Reference	
<41.1 g/L	1.601 (0.738, 3.477)	0.234
PNI, *n* (%)		
>48.4	Reference	
≤48.4	3.952 (1.742, 8.964)	0.001^#^

TNM stage, tumor node metastasis stage; WBC, white blood cell; ASA, American Society of Anesthesiologists; PNI, prognostic nutritional index. ^#^Indicating statistically significant (*p* < 0.05).

## 4 Discussion

### 4.1 Interpretation of main findings and comparison with previous studies

This study indicated that preoperative PNI served as an independent risk factor of POD in GC patients. Our findings aligned with previous reports establishing PNI as a reliable predictor of postoperative complications across various cancer types (24–28). ROC analysis confirmed the discriminative capacity of PNI, highlighting its clinical utility for POD risk stratification, consistent with studies documenting comparable predictive performance of PNI for clinical outcomes in oncology populations (27, 29–31). Up to date, several studies have explored the relationship between preoperative PNI and POD risk in cancer patients (Supplementary Table 1) (20–22, 32–35). Tei et al. (20) from Japan initially identified PNI as an independent predictor for POD in CRC patients, though their subsequent research yielded conflicting results (35). Similarly, Mokutani et al. (32) reported no significant association between PNI and POD risk in CRC patients. A following study in Korea revealed that delirium occurred more frequently in the low PNI group among lung cancer patients (34). Nakamura et al. (33) from Japan suggested that PNI did not vary among complication and non-complication groups, though the relationship between preoperative PNI and the risk of POD remained unreported. A Chinese study by Shen et al. (22) showed that preoperative PNI was significantly associated with the risk of POD in esophageal cancer patients, which was consistent with our previous study (21). Notably, a recent meta-analysis concluded that preoperative PNI showed no significant relationship with POD risk (36). We postulated that the limited sample sizes and substantial clinical heterogeneity may account for this negative association (36). Importantly, no previous studies have specifically explored the relationship between preoperative PNI and POD risk in GC cases. In this study, we firstly reported that preoperative PNI was an independent predictor for POD among GC cases. Totally, we observed that the associations between preoperative PNI and POD risk among different cancers were diverse. The following points may explain it. One, the sample sizes of different studies were distinct. Two, the cut-off values of PNI varied. Three, different cancers and surgical methods (minimally invasive versus open procedures) were also potential reasons. Collectively, our findings provide preliminary evidence supporting PNI’s utility as a predictive biomarker for POD in GC patients.

### 4.2 Possible mechanisms linking low PNI and POD occurrence

Delirium and malnutrition were identified as mutually reinforcing, and nutrition strategies were recognized as valuable interventions for delirium management (37). The pathogenesis of delirium involved multiple mechanisms including cerebrovascular dysfunction, neurotransmitter imbalances, metabolic disturbances, and impaired neuronal network connectivity (12). Malnutrition represented a well-established marker for delirium (38). Malnutrition could cause a variety of changes including immune dysfunction, electrolyte imbalances, and the alterations of drug metabolism, which may contribute to the pathogenesis of delirium (38). A recent meta-analysis reported that preoperative malnutrition was significantly associated with the risk of POD (39). As a composite nutritional marker, PNI reflects both nutritional and immune status through serum albumin and lymphocyte count. Hypoalbuminemia can compromise the integrity of the blood–brain barrier, impair detoxification processes, disrupt metabolic disorders of brain cells, and amplify neuroinflammatory responses, which may associate with cognitive dysfunction (8, 40, 41). Concurrently, reduced lymphocyte counts indicated impaired cellular immunity, predisposing patients to systemic inflammation and infection, recognized precipitants of delirium (11, 42). Additionally, surgical stress from GC resection promotes proinflammatory cytokine release, potentially disrupting blood–brain barrier function and neuronal signaling (43, 44). The underlying mechanisms of association between PNI and POD among GC patients warrant further studies.

### 4.3 Other risk factors for POD

Beyond PNI, we identified additional independent risk factors for POD. Our data indicated that advanced age (≥60 years) and higher ASA classification demonstrated significant associations with increased POD risk in GC patients. Delirium was a multifactorial syndrome affecting older patients. Patients with advanced age are at higher risk of delirium (45). Older age is recognized as a risk factor for delirium (8). In this study, we also found that age >60 years was a risk factor for POD, which was consistent with previous studies (46–48). Additionally, we found that high ASA score was associated with an increased risk of POD among GC patients. Our results regarding ASA classification aligned with those reported by Liu et al. (48).

### 4.4 Clinical implications and recommendations

PNI represents an inexpensive, readily accessible parameter that could be incorporated into routine preoperative assessment to identify GC patients at elevated POD risk. This identification enables targeted interventions including nutritional optimization and enhanced perioperative monitoring. Patients with low PNI may benefit from multidisciplinary prehabilitation involving dietitians, physiotherapists, and geriatric specialists, potentially reducing POD incidence.

We recommend integrating PNI into risk stratification instruments or predictive nomograms to assist surgical and anesthesia teams in developing individualized care plans. Active screening and nutritional interventions may alleviate POD burden, improve surgical outcomes, and reduce healthcare expenditures (7). Interestingly, emerging evidence suggested dexmedetomidine may reduce delirium incidence (49–51). Key challenges in POD management included: (1) developing effective methods for identifying high-risk patients, and (2) establishing personalized nutritional therapies and effective pharmacological interventions for malnourished patients. These areas require validation through prospective clinical trials.

### 4.5 Limitations of the study

There were a few limitations that need to be admitted. First, the single-center retrospective design showed potential selection bias and may limit generalizability. Second, despite adjusting for multiple clinical factors, residual confounding from unmeasured variables—including specific medications, pain management strategies, and psychosocial factors—may persist. Third, we did not assess serial PNI measurements or postoperative nutritional interventions’ impact on POD development. Fourth, other nutritional indicators should also be included in this study, which is beneficial for a more comprehensive evaluation of the preoperative nutritional status of GC patients. Fifth, the sample size of this study was not large enough. Especially in this study, many variables were included, which made some conclusions not trustworthy. Finally, the underlying mechanisms of association between PNI and POD among GC patients were not investigated.

## 5 Conclusion

This study establishes preoperative PNI as an independent predictor for POD in GC patients. PNI serves as an efficient preoperative screening tool to identify high-risk individuals, enabling early preventive measures. Future research should evaluate the potential benefits of nutritional support in preventing POD and improving long-term patient outcomes.

## Data Availability

The raw data supporting the conclusions of this article will be made available by the authors, without undue reservation.
