# Supplementary material for: Association of preoperative prognostic nutritional index with postoperative delirium after gastric cancer surgery
Source: Front Nutr. 2026 May 20;13:1817605. doi: 10.3389/fnut.2026.1817605 (PMC13229701; doi:10.3389/fnut.2026.1817605)
Supplement: Supplementary file 1 [file Table_1.docx]

Supplementary Table 1. The association between preoperative PNI and the risk of POD among cancer patients.

| **Author** | **Year** | **Country** | **Types of cancers** | **Rate of delirium** | **Cut-off value of PNI** | **Association of PNI with POD** |
| --- | --- | --- | --- | --- | --- | --- |
| Tei et al. | 2010 | Japan | Colorectal cancer | 10.9% | Unreported | Yes |
| Tei et al. | 2015 | Japan | Colorectal cancer | 14.1 % | Unreported | No |
| Mokutani et al. | 2016 | Japan | Colorectal cancer | 21.8% | Unreported | No |
| Park et al. | 2020 | Korea | Lung cancer | 6.4% | 50.0 | Unclear* |
| Nakamura et al. | 2025 | Japan | Colorectal cancer | 19.3% | 40.0 | Unclear# |
| Chen et al. | 2025 | China | Esophageal cancer | 16.99% | 50.9 | Yes |
| Shen et al. | 2025 | China | Esophageal cancer | 35.4% | Unreported | Yes |
| Hong et al. (This study) | 2025 | China | Gastric cancer | 29.1% | 48.4 | Yes |

PNI, prognostic nutritional index; POD, postoperative delirium

*This study only showed that delirium occurred more frequently in the low PNI group (PNI < 50.0)

# This study indicated that PNI did not vary among complication group and non-complication group.
